# Sensory Evaluation and Consumers’ Acceptance of a Low Glycemic and Gluten-Free Carob-Based Bakery Product

**DOI:** 10.3390/foods13172815

**Published:** 2024-09-05

**Authors:** Luigi Esposito, Nicola Casolani, Marco Ruggeri, Umile Gianfranco Spizzirri, Francesca Aiello, Emilio Chiodo, Maria Martuscelli, Donatella Restuccia, Dino Mastrocola

**Affiliations:** 1 Department of Bioscience and Technology for Food, Agriculture and Environment, University of Teramo, 64100 Teramo, Italy; lesposito2@unite.it (L.E.); ncasolani@unite.it (N.C.); echiodo@unite.it (E.C.); dmastrocola@unite.it (D.M.); 2 Department of Management, Sapienza University of Rome, Via del Castro Laurenziano 9, 00161 Rome, Italy; m.ruggeri@uniroma1.it; 3 Ionian Department of Law, Economics and Environment, University of Bari Aldo Moro, 74123 Taranto, Italy; umile_gianfranco.spizzirri@unical.it; 4 Department of Pharmacy, Health and Nutritional Sciences, University of Calabria, 87036 Rende, Italy; francesca.aiello@unical.it

**Keywords:** food choice, gluten-free, carob, consumer acceptance, glycemic index

## Abstract

Carob pulp flour has antidiabetic and antioxidant activities, is naturally sweet, and is rich in fibers. It is obtained from carob pod pulp from the evergreen tree *Ceratonia siliqua* L., which is grown in Mediterranean areas and is known for locust bean gum production. Despite its valuable effects on health, such as the modulation of the glycemic index, this ingredient has a tremendous impact on technological and hedonic features, mainly on color, flavor, and texture. In this paper, the qualitative features and consumers’ acceptance of a carob-based gluten-free bakery product where rice flour was substituted at 40% with carob pulp flour were studied. A panel group of experts described the bread as dark, quite dense, sweet, aromatic, and with a limited bubble dispersion. On the other hand, the sensory assessment and the willingness to pay of consumers were assessed in two groups (a fully informed one about heathy attributes of the food and a blind one). The results indicated a moderate appreciation of the overall quality of the product (average score between 4 and 5 points on a 9-point Likert scale). The information about the food’s healthy properties and the ability to maintain a low glycemic index did not enhance the consumers’ perception of the product, while previous knowledge and involvement in the product consumption were perceived to have primary importance regarding the final consumers’ choice. Finally, an accelerated shelf-life test was run on the packaged snack to evaluate the general quality and stability. The protective packaging helped in limiting bread decay and maintaining the textural characteristics.

## 1. Introduction

Low-glycemic products are becoming more and more popular among consumers due to their high level of convenience. Goods formulated within this scope must combine the desired hedonic characteristics of distinct products with an improved recipe that reduces the use of or substitutes for specific ingredients [1].

People suffering from diabetes (type 1 and 2), those with other non-communicable diseases, or those at risk of developing these conditions must be very aware of the glycemic index (GI), and thus, modulating the intake of carbohydrate-containing food which may raise blood glucose levels quickly, moderately, or slowly is important [2]. The GI control must become a common practice for all, but especially for celiac subjects and generally for people with high sensitivity to gluten, since often, alternative available goods employ medium- to high-GI ingredients to substitute for wheat and other cereals [3]. This is particularly true for bakery products, which are very popular and sometimes difficult to reformulate [4]. The gluten removal poses several nutritional issues. In fact, when comparing gluten-containing bakery foods with their gluten-free (GF) counterparts, the latter usually show higher levels of fats, carbohydrates, sugars, fermentable oligosaccharides, disaccharides, monosaccharides, polyols (FODMAP’s), and sodium [4]. Moreover, the replacement of wheat with rice or corn flours can result in fiber, protein, folate, iron, potassium, and zinc deficiencies, as well as a higher GI [2]. In this sense, carob pulp flour could be considered a valuable substitute for rice flour as it possesses several nutritional advantages, showing high quantities of fiber, potassium, phosphorus, protein (caroubin), and inositols, as well as cocoa aroma compounds (furans, esters, and pyrroles). Moreover, it possesses many health-promoting effects, including mitigation of gastrointestinal diseases, diabetes, hyperlipidemia, inflammation, and oxidative stress [5]. Finally, carob pulp flour has a much lower GI than rice flour. In this regard, Restuccia et al. (2023) [6] reported that a 40% replacement of rice with carob pulp flour reduced the GI of a GF baked product by a significant amount in comparison to the control, while lower levels of replacement did not produce any significant effect.

Generally, the consumer knowledge about the effects and risks of high-GI products is not so clear. As reported by Barclay et al. (2021) [7], in Canada and the European Union, GI claims are not legal under current food laws, while in other countries, labels are completed with the GI information of the goods. Thus, consumers in many Western countries are not supported in their dietary choices, even if these populations are the most vulnerable to diabetes, obesity, cardiovascular disease, and other syndromes [8]. At any rate, when proposing a new product, several evaluations regarding people’s perception and expectations must be carried out to gain knowledge about product substitutes.

Consumer acceptance of a new product is a multifaceted process that is crucial for market success [9], and involvement in health aspects of product consumption can play an important role in this process. Involvement is a motivational construct widely analyzed in the literature [10], and it deals with the personal relevance given by consumers to a product choice and its consumption. It is correlated to self-reported knowledge, but not necessarily to the objective one [11], and has effects on consumers’ decision processes and purchase behavior [12]. Food consumers’ perception is heavily influenced by health information, which can drive acceptance and consumption patterns. In fact, one of the main motivations reported in the scientific literature for food consumer acceptance is health [13,14]. Many studies have analyzed the effects of information on some products’ characteristics or health benefits on their sensory evaluation and the willingness to pay (WTP) of consumers [15,16,17]. The role of health aspects in shaping consumers’ perceptions has become increasingly prominent in recent years, driven by a growing awareness of the relationship between diet and well-being. The rising prevalence of lifestyle-related diseases such as obesity, diabetes, and cardiovascular conditions has intensified the focus on healthful eating, encouraging consumers to prioritize foods with functional and nutritional benefits [18]. The review of Topolska et al. (2021) [14] analyzes consumer attitudes towards functional foods, aiming to better understand their needs and behaviors related to such products. The research indicated that nutritional knowledge is one of the main drivers, and claims about the disease-preventive properties of functional foods are particularly appealing to consumers. Additionally, it emerged that women are more inclined to balance taste with health benefits. Plasek et al. (2020) [19] found only a small portion of respondents were unwilling to spend money to improve health issues. Studies have shown that health claims on food products can significantly impact consumers’ choices, as individuals increasingly seek foods that contribute to their overall health and wellness [20,21]. Effective communication strategies highlighting benefits can help to overcome barriers related to taste and unfamiliarity, thus facilitating greater acceptance and consumption [16]. Consumers’ prior understanding and familiarity with food characteristics or ingredients can greatly influence the appreciation of new products [22]. Research indicates that informing consumers about a product’s health advantages and ingredients generally boosts its perception and sensory evaluation [15,16].

Few studies have examined the perception of carob-based foods, and most are recent investigations [23,24,25]. Caliskan et al. (2023) [25] discovered that consumers are inclined to purchase carob bars as a substitute for chocolate bars. Papageorgiou et al. (2020) [23] evaluated the perception of cakes made with carob flour, finding that incorporating 30% and 50% carob flour produces cakes with acceptable texture and sensory qualities comparable to cakes with 20% cocoa. García-Díez et al. (2022) [24] studied a cocoa–carob blend and found that the sensory analysis indicated that it is well-received by consumers, especially when information about its potential biological benefits is provided, leading to a significant improvement in taste evaluation. The interest in the science of carob pods relies on several factors involving both food formulation, cultural aspects, and ethical environmental issues [26] Carob pods have been used for centuries in the entire Mediterranean basin in different ways, such as animal feed. These fruits are part of the traditions and cultural identity of these countries, often being linked to scarcity and poverty [26]. While the pulp is culturally considered as a livestock commodity, or an emergency food for humans, the seeds have an established and precious economic chain, being a resource for locust bean gum, a natural thickening ingredient largely used as additive worldwide, which is identified with the code (E-410) [27]. Furthermore, carob seeds were historically used as unity of measure for gold and diamonds. The karat from the Arab name of the carob tree “*quirat*” is still used for the same reason [5]. The cultivation of *Ceratonia siliqua* L., besides suffering a decline in last decades, is now experiencing a revival due to its resilience against fast climatic changes and the urgent hydric crisis [28]. The pulp of this fruit is also gaining growing importance as a rediscovered ingredient with high-value properties. In particular, the relevant content of proteins, the quality of carbohydrates, the sweet and cocoa-like natural taste, and the interesting levels of fibers and minerals make this ingredient attractive for several applications in food formulations and/or ingredient substitutions [5]. As a matter of fact, carob pulp flour (CPF) has found implementation in cocoa-based baking and other products [29,30,31]. Among these, gluten-free and reduced-sugar bakery products may be special candidates due to the fast development of defective features such as firming, increased crumbliness, and discoloration [32], while compromising leavening, the textural properties, and the correct structure development [33].

In addition, CPF has flavoring and coloring features that may positively or negatively influence consumers’ perception. It contributes to an increase in the amount of antioxidant species, especially phenolic acids and flavonoids [6]. Some negative traits may also come out, such as the brown color; the typical flavor, which is often described as cheesy; and the presence of fibers interfering with the texture.

On one hand, it examines the promising impact of CPF as a coadjuvant in gluten-free formulations, considering additional health benefits such as the mitigation of the GI. On the other hand, it addresses the challenges in obtaining a product with proven health benefits, also taking into account the technological properties, processing constraints of CPF inclusion, its impact on shelf life, and an in-depth analysis of consumer acceptance and perception. All this information put together represents the novelty of this study, especially when compared with the available scientific literature on the topic.

## 2. Materials and Methods

### 2.1. Carob Flour-Based Bakery Product: Preparation and Sampling

The bakery product was obtained following the recipe and procedure described in our previous work [6], then was packaged according to a commercial procedure. Each loaf (180 g) was individually packaged after complete cooling at room temperature in coextruded polypropylene (PPCX 30 μm, 27.3 g m^−2^, Poplast srl, Castel Sangiovanni, Piacenza, Italy) film; the packages were hermetically sealed (MOTOKRIMPER, NAIS, Milan, Italy). The obtained breads were packaged, and a part was used for the consumer test (within two days of packaging, see Section 2.6). The rest was subjected to the accelerated shelf life (ASL) test, as described below. The trial was replicated on two independent occasions.

### 2.2. Accelerated Shelf Life (ASL) Test

To evaluate the stability of baked products, a test of accelerated shelf life was carried out. Packaged samples were stored at 40 °C and controlled R.H. (55%) with no light for 30 days in an Incubator model (FOC 225 E, Velp Scientifica Usmate Velate MB Italy). The sampling for the analyses was performed immediately after preparation (0 days, t_0_); after 3 days (t_3_), 7 days (t_7_), and 14 days (t_14_); and at the end of period of observation (t_30_) in ASL conditions.

### 2.3. Moisture Content and Water Activity (a_w_)

Moisture content was measured according to the gravimetric AOAC 925.09 method (AOAC, 2005) [34] using an oven method at 105 °C for 12 h.

The values of water activity (a_w_) were obtained with the Aqualab 4 TE kit (Court Pullman, WA, USA).

All values were measured in triplicate, and the results are expressed as the mean and standard deviation.

### 2.4. Physical Analysis

Color was determined by a colorimeter CR-5 (Spectrally based, Konica Minolta, Tokyo, Japan) with a D_65_ light source and a 10° observer. The analysis was carried out with the slices of samples in different locations (at least nine specimens at each time of sampling). Color was expressed using L* (lightness, intensity of white color), a* (+*a*, red; −*a*, green), and b* (+*b*, yellow; −*b*, blue) values. The coordinates a* and b* were used to calculate the hue angle value [=arctan (b*/a*)] and the chroma or saturation index [=(a*^2^ + b*^2^) ^1/2^].

Mechanical properties were evaluated by modifying the method reported in [35] Martuscelli et al. (2008) using a dynamometer Instron mod. 5542-H5036 (Instron International Limited, High Wycombe, UK) equipped with a maximum-load camera of 500 N.

Round slices of bread were obtained from the packaged loaves. The thickness was 0.15 cm and the diameter was 4 cm. The analysis (at room temperature, 22 ± 2 °C) was executed using a flat shear blade probe, and ten specimens were tested for sampling.

Maximum force (F_m_), expressed in N, was used as the index of the maximum hardness (Bourne, 2002) [36]; the reciprocal of the Et/F_m_ ratio, i.e., the index of the degree of deformability, was used as the *friability index* (mm^−1^) [35]).

### 2.5. Descriptive Sensory Analysis

Samples were evaluated by means of quantitative descriptive analysis (QDA) for five classes of descriptors, grouped as: appearance, odor, flavor, taste, and textural properties. All terms for describing samples were evaluated and chosen according to a recent literature review [23,37]. A panel group of five women and four men ranging from twenty-five to fifty years old (28.2 years was the average age of the panelists) was trained to conduct a quality assessment of the gluten-free bakery products. Thus, panelists were recruited after their informed consent was collected and a general consensus was reached on data treatment and privacy protection according to the current European law (GDPR 2016/679) [38]. Then, their ability to distinguish odors and tastes was evaluated. Finally, panelists were trained for vocabulary development through a series of triangular tests as reported in ISO 8586:2023 [39]. The training duration was 80 h, including familiarization with relevant descriptive terms and the use of intensity scales (ISO) 4121:2003 [40].

According to Magano et al. (2022) [41], specific training was given on how to conduct the training of the panelists and on the questions to deliver. Celiac and non-celiac subjects did not differ in their sensory descriptions of the gluten-free bread, and their preference was based on the same sensory attributes [42]. Panelists were asked to taste a slice served on a white dish. Panelists were provided with individual templates, where the descriptors were grouped per section. Intensity scales were used; the values ranged from 1 to 5; 1 represented the absence of the attribute, while 5 represented the maximum. Samples were obtained from loaves (180 g) cooked on the same day as the sensory test (t_0_) or packaged and stored in ASL condition at the end of the observation time (t_30_). Sensory tests and training sessions were carried out in the sensory laboratory of the University of Teramo, which fulfilled the required standards for these analyses according to ISO 8589:2007 [43].

### 2.6. Consumer Test

The liking and willingness-to-pay (WTP) for the carob flour-based product were analyzed through a consumer test composed of a questionnaire and a sensory assessment (within two days of production and packaging) provided to two different groups of consumers. While the questionnaire was common to both groups, the sensory assessment and the WTP were assessed using different levels of information: under blind conditions (without information on the characteristics of the tasting product) for the first group and under full-information conditions (complete description before tasting of the product ingredients and of the positive effects of carob-based products on nutrition and health) for the second one.

The questionnaire was posed in a general way regarding bakery products’ consumption, and it consisted of: informed consent and authorization for data treatment and protection in accordance with the current European law [38] demographic variables; questions concerning consumer involvement in food product consumption; questions concerning consumer knowledge of specific health aspects of food consumption; knowledge and consumption of products containing carob flour; and consumption habits regarding some bakery products.

In this way, it was possible to compare the perception of the product’s quality with the psychographic characteristics of the respondents, combining the demographic variables with product knowledge and involvement in product consumption [12,44].

The demographic variables considered were age, gender, level of education, and financial situation. The last category was divided into good, normal, modest, and difficult based on respondents’ self-perception of their economic status, which is a recognized indicator of consumer behavior, as the perceived financial situation can influence the willingness to buy products [45].

As far as involvement is concerned, the respondents were asked to address four questions using a 1–5 Likert-type scale (from 1: completely disagree to 5: completely agree), as shown in Table 1.

Involvement was calculated as the sum of the scores of the four questions, and respondents were divided into two groups [46]: “low values” (the score was below the mean) and “high values” (the score was above the mean).

The evaluation of consumers’ knowledge was focused on health aspects of food consumption. It was measured according to the knowledge of the meaning of the glycemic index and the respondents’ capacity to provide examples of high-/low-glycemic-index products.

In order not to affect the first group’s liking and WTP of the product (blind), the questions were generically focused on bakery products.

The awareness of carob flour as an ingredient (“Do you have any knowledge of carob flour?”) and prior consumption of products produced with carob flour (“Have you ever tasted products containing carob flour?”) were also tested.

The liking of and WTP for the product were analyzed for the two groups of respondents: under blind conditions and with full information.

The text presented for the full-information tasting was the following:

*“The operator will give you now for tasting a bakery product, produced with the following ingredients: rice flour, carob flour, cornstarch, sourdough, kefir and salt. The carob-based products are a good source of food fibers, sugars and a set of bioactive compounds as polyphenols. The bioactive compounds present in the carobs help controlling numerous health problems, such as diabetes, cardiovascular diseases and gastrointestinal disorders, thanks to their anti-hyperglycemic, anti-oxidant and anti-inflammatory activities.”*

The survey was managed by alternately providing a questionnaire containing or not containing information about the product characteristics to the participants, so that they were randomly assigned to one of the two groups.

The liking was measured on a 1–9 Likert-type scale (from 1: extremely bad to 9: excellent) The participants were asked to answer the following question:

*We kindly ask you to taste the following sample and classify according to your judgment the overall quality of the product you have tasted (1—Extremely bad; 5—Neither good nor bad; 9—Very good).*

The WTP was measured on a scale from 0 to 6 euros for one kilogram of the tasted product through the following question:

*How much would you be willing to spend for a kilogram of the product you just tasted?*

Following Ferreira et al. (2021) and Botelho et al. (2017) [47,48], the absence of statistical differences among the respondents’ profiles of the two groups (blind and full information) allowed for a comparison between them regarding liking and WTP. Demographic variables were used for the comparison, such as age, gender, study title, and economic situation.

### 2.7. Statistical Analysis

For the technological results, statistical significance was assessed using analysis of variance (ANOVA) and the LSD (least significant differences) test, and multiple comparison analysis was conducted by means of XLSTAT software, version 2019.1, for Microsoft Excel (Addinsoft, New York, NY, USA). All results were considered statistically significant at *p* < 0.05.

Statistical analysis of consumer test data was conducted using SPSS 26.0. Differences between groups were compared using *t*-tests and Pearson’s chi-square tests. Results with *p*-values less than 0.05 were considered statistically significant.

## 3. Results

### 3.1. Quality and Sensory Attributes of the Carob-Based Bakery Product

**Figure 1:**
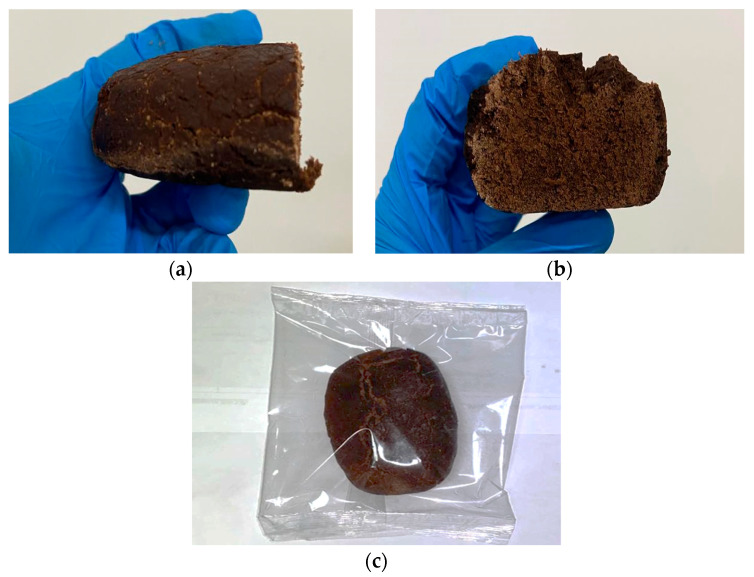
Carob flour-based bakery product immediately after preparation. Internal and cross-section detail (**a**,**b**). Packaged as whole product (**c**).

A general characterization of the freshly baked product (Figure 1a–c) was performed to assess the main features. Moisture, water activity (a_W_), and colorimetric and textural properties are reported in Table 2. CPF-based bread resulted in 33.1 ± 0.26 for moisture (%) and water activity (a_w_) of 0.927 ± 0.001. Concerning color attributes, our bread had a uniform brown coloration, slightly lighter on the crust. Dynamometric analysis assessed the texture properties, recording the values of hardness and friability (161.3 ± 30.6 N, 0.26 ± 0.07 mm^−1^, respectively).

From Table 3, it is visible that the panel test analysis (QDA) qualified the obtained bread (T_0_) as dark, quite dense, sweet, and aromatic. As a matter of fact, the panelists scored the bread as 4.89 ± 0.33 on a 5-point scale. The panelists found limited bubble dispersion. The odor characteristics described for our bread stated that it resembled rye-based products, and defective attributes such as beans had a low score. Also, any other marker for an off odor was indicated by the panelists when asked. The taste, as anticipated, was not remarkably specific, but the sweet sensation was the main aspect recorded by panelists. Regarding flavor, cocoa had the highest value of 3 ± 1.66. Other attributes, despite having lower values, maintained discrete variance, confirming the complexity of CPF. The texture properties were described as medium-hard and chewy, with limited crumbling; this is directly linked to the CPF fiber content, influencing the density and cohesiveness of the final product.

### 3.2. Effect of the Accelerated Shelf-Life Test on the Stability of Technological Characteristics of Carob Pulp Flour-Based Bakery Product

**Figure 2:**
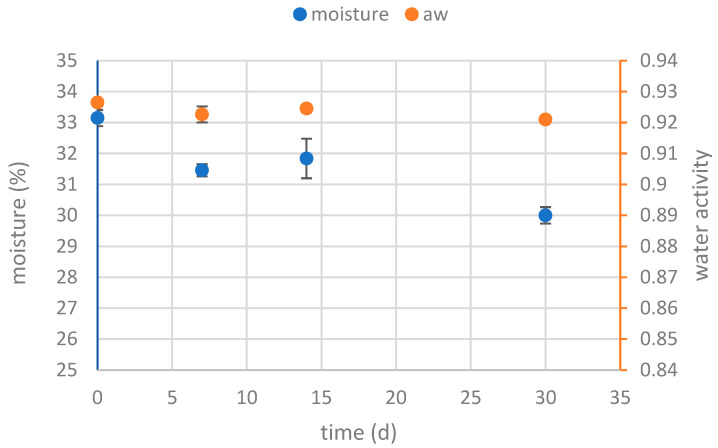
Results of moisture and water activity trend during packaged storage, in ASL conditions (40 °C, no light).

In the graph in Figure 2, a_W_ and moisture trends during the ASL test conducted on packed breads are reported. It can be noticed that limited moisture was lost, the last product analyzed remained quite equal to the freshly baked bread. The final moisture was just 2.85%.

As is visible from Table 4, the color characteristics changed over time, with a small but continuous decrease for all coordinates. In particular, a* (redness when positive or greenness when negative) and b* (yellowness when positive or blueness when negative) were significantly different (*p* < 0.001). Our results described lighter breads at the end of the storage. We noticed changes in the crust: the lightness at the end of the storage (T_30_) went from 23.29 ± 0.8 to 31.56 ± 2.15, and the chroma was increased as well, along with the hue angle, thus resulting in more saturated and vivid color. 

Concerning the sensory analysis, in this paper, we compared T_0_ vs. T_30_ samples (Table 3) for their general appearance, odor, flavor, taste, and texture. We adopted a 5-point scale and asked trained panelists to score the attributes found in previous training sessions. The two samples were thus evaluated, and significant differences were found. Panelists perceived the bread color to be lighter; in particular, the crust was lighter (*p* < 0.05). Taste characteristics remained quite unvaried except for sourness, which was perceived as higher (*p* < 0.05) at the end of the storage. Hardness and crumbling remained quite unaltered, while chewiness decreased statistically significantly (*p* < 0.05).

### 3.3. Consumer Test Results

#### 3.3.1. Sample Description

The consumer test was carried out at the University of Teramo with voluntary participants, resulting in a convenience sample composed mainly of university students (75%) and personnel (16%). The number of respondents was 102, with mostly females (63.7%) over males (36.3%). The average age of the respondents was 26.8 years, with the youngest participant aged 19 and the oldest aged 54.

The education level was high, with all the participants holding at least a high school degree: 51% undergraduate (almost all university students), 24.5% bachelor’s degree, and 24.5% master’s degree.

Among students, different study courses were represented so that any possible bias connected with studies in food science or similar was avoided. Considering the financial situation, many of the respondents perceived their economic status as good (18.6%) or normal (61.8%).

The socio-demographic characteristics of the sample are reported in Table 5. From the comparison of the statistical differences between the respondents’ profiles of the two groups, no significant differences emerged in any of the considered variables, indicating strong similarity between the two groups.

#### 3.3.2. Acceptance and WTP: Effects of Information

The results of the sensory test (Table 6) indicated a moderate appreciation of the overall quality of the product, which received an average score between 4 and 5 points on a 9-point Likert scale (average score 4.53, standard deviation 2.08), where 1 represents “Extremely bad” and 9 represents “Excellent.” The average willingness to pay (WTP) was EUR 1.92/kg (standard deviation 1.63).

Concerning the product’s suitability, 38.2% of respondents considered it more appropriate for breakfast, 30.4% for a snack, and 28.4% as an accompaniment to lunch or dinner. Only 2.9% found it suitable as a dessert after meals.

Given the absence of significant socio-demographic differences between the two sub-groups interviewed, it is possible to compare the assessments of liking and WTP between consumers who tested the product without information about its composition or properties and those who received full information. The group that assessed the product without information gave an average score of 4.75, while the informed group gave a lower average score of 4.30. The WTP was EUR 1.94 for the uninformed group and EUR 1.90 for the informed group.

The results show a lack of a significant difference in the product’s perception between the two sub-groups (Table 6). It implies that the information provided did not improve the product’s perceived sensory appeal or value and did not enhance the perception of the product with better characteristics in terms of composition and health effects. In this case, the information did not seem to alter the preferences.

#### 3.3.3. Acceptance and WTP: Effects of Knowledge

Since the information did not modify the sample preferences, both the knowledge about the glycemic index and the awareness of carob flour as an ingredient were analyzed across the entire sample. To assess respondents’ knowledge about the glycemic index, a specific question was posed, presenting two statements with opposite meanings:

*“Glycemic index is a value that expresses the speed with which food containing carbohydrates increases the concentration of glucose in the blood/the slowness with which food containing carbohydrates decreases the concentration of glucose in the blood.”*

In total, 91% of respondents chose the correct answer. Participants were then asked to provide examples of products with high and low glycemic indices. In this case, only 49% provided correct examples of both types of products. Participants who answered correctly about the glycemic index and provided accurate examples were considered to have correct knowledge about the topic.

Next, participants were asked if they had any knowledge of carob flour and if they had ever consumed carob flour-based products. Respondents were equally divided regarding carob flour knowledge (49% yes, 48% no, 3% do not know), but only 14% were aware of having consumed carob flour-based products (24% did not know and 52% had never).

Both consumers’ knowledge of carob flour as a food ingredient and their understanding of the glycemic index, measured by their ability to provide examples of high and low glycemic index products, seemed to increase the tested product appreciation and WTP, as indicated in Table 7 and Table 8. A statistical difference in the WTP (*p* < 0.05) was observed for consumers already familiar with carob flour, who showed a higher willingness to pay for the product compared to those who had never heard of it before. These results highlight that it was not the information provided at the time of the experiment that modified consumers’ preferences, but rather their prior knowledge. The study’s findings suggest that educational interventions aimed at increasing consumer knowledge about specific health benefits of products and ingredients could be more effective than mere labeling or in situ information provision.

#### 3.3.4. Acceptance and WTP: Effects of Involvement

Involvement in the health aspects of food consumption was associated with a high interest in the entire sample regarding the health and composition aspects of the products in terms of ingredients. However, the specific topic of the glycemic index received less attention, with values below 3 (see Table 9). As mentioned above, consumer involvement was calculated as the sum of the scores from four questions:

*“I consider myself a consumer that pays attention to health aspects; I carefully read labels; I pay attention to the composition of the product in terms of ingredients; I mind the glycemic index.”*

Respondents were then divided into two groups (following [46]): “low involved” (score below the mean) and “high involved” (score above the mean). Statistical differences emerged in both liking and WTP (*p* < 0.05) between consumers involved and not involved in health aspects. In both cases, values were higher for the highly involved group (see Table 10).

## 4. Discussion

As noted in previous studies [6,49], CPF is rich in fibers (especially insoluble), increasing the cohesiveness of products. This characteristic directly influenced the final moisture of the investigated bread. It was found that adding up to 30% of CPF to wheat flour for bread production resulted in less moist breads [49], while values around 10–20% led to similar or higher moisture (in respect to the control) [49,50]. With respect to water activity (a_w_), typical values of common wheat-flour breads fall into a range around 0.93–0.96 according to specific recipes and production protocols [51]. Similarly, to mimic these goods, gluten-free formulations employ sugars, gums, lipids, and other ingredients. Very often, lower a_w_ values are found (0.89–0.93) due to different leavening processes, such as the use of baking powder instead of yeast. Our sample is in line with gluten-free breads, and the short list of ingredients within the absence of any additive encourages the use of CPF as a positive technological ingredient which is more than functional. Concerning color attributes, similar or lower values, especially for the brightness (L) parameters, may be found in the literature for common products such as rye and whole-grain breads [52]. The textural properties of our good are not very different from other gluten-free breads; they denser and less spongy, as assessed by Sciarini et al. (2012) [53]. Tsatsaragkou et al. (2017) [54] found that the incorporation of CPF in a certain ratio (15:85) produces harder and less moist crumbs, but the bread is less prone to becoming firmer during the storage period. Moreover, the moisture loss measured on the crust is lower than in commercial gluten-free breads. Aside from the specific features of CPF, the addition of CPF with a coarse particle size and the use of the jet milling technique for reducing the carob flour particle size could lead to end products with reduced firming rates compared to commercially available samples. The study of Papageorgiu et al. (2020) [23] showed that the inclusion of up to 30% CPF as a cocoa replacer in cakes leads to reduced hardness, decreased springiness, and cohesiveness. Besides the employment of kefir as leavening agent, along with certified gluten-free yeast, rice flour and corn starch are not capable of creating a stable network for maintaining CO_2_ bubbles in the loaf. In addition, the limited rise in volume during the leavening and the cooking processes has a direct impact on the final texture, the faster development of staling, and crumbling. This condition pushed us to evaluate the qualitative decay of our bread in a protective packaging.

The hardness and friability were slightly higher than those of similar products where rice flour was the principal ingredient [55]. It is important to highlight that these values of hardness are ascribable to the richness of fiber constituents of CPF, which strengthen the structure of dough formulations [56]. Similar observations on dynamic rheological studies have been reported for wheat dough supplemented with carob fiber [57]. The high percentage of soluble sugars in carob powder, especially fibers, and their melting and interaction with the rest of the ingredients could lead to stronger interactions during baking process [58,59,60]. The effect of sugar is unclear and depends on particle size and crystalline form [61]. Meanwhile, fiber, although its effect also depends on its type, particle size, and morphology, generally reduces the expansion of the dough and increases its hardness [62].

The primary action of CPF concerns its coloring capacity. CPF reduces lightness and increases redness; this ability comes from the roasting degree and the occurrence of Maillard reactions [63]. The homogeneous distribution of the color is also related to the particle size; the finer the particle size, the greater the darkness. As previously reported, CPF is indicated as a cocoa replacement, finding plenty of applications, especially in bakery products [64]. With the relatively high percentage of CPF tested herein, the panelists found a limited bubble dispersion that was higher than what was found in the full rice flour-based products assessed in our previous work [6]. The discrete variance in flavor attributes can be attributed to the complexity of CPF. In fact, the volatile profile of CPF includes several acids, esters, aldehydes, ketones, and alcohols, which are strongly influenced by the ripening stage and roasting degree [65]. Texture properties (density and cohesiveness) are directly linked to the CPF fiber content; on one hand, the higher degree of purity of the CPF leads to a major presence of soluble polysaccharides and sugars; on the other hand, if residual carob seed powder is present, the cohesiveness and density are highly increased [66]. In a study on high-proteins bread [67], it was shown that the inclusion of CPF (+15%) was responsible for the formation of a secondary network between non-wheat proteins. This behavior ameliorates the general performance of the final bread, leading to increased volume and reduced hardness.

The protective effect on the moisture loss may be primarily ascribed to the plastic bag used. As previously reported, fibers and polysaccharides of CPF may act as plasticizers, binding the water. The same considerations can be drawn for the a_W_ behavior. In general, limited interactions with the external environment occurred, and a limited redistribution of water was registered with time. Considering the intense stress conditions of the ASL test, we can define the product as being highly stable and not prone to becoming stale with common values of temperature and moisture conditions. Moreover, the type of packaging used in this study is a primary type; thus, a second layer of protection (i.e., against the light) may be imagined, such as a secondary packaging.

Regarding color attributes, they have a primary role in food acceptance, especially for intensely colored products. In the case of cocoa, for example, the intense brown color is recognized by common consumers as a positive trait confirming quality. In a recent work by Pawłowska et al. (2018) [37], the authors noticed reduced changes in the color parameters of muffins when cocoa was replaced by CPF. Despite few differences in the crumbs, the general aspects of cocoa and CPF muffins were not perceivable by consumers. Of course, in cocoa powders production, technological processes play a role changing its final color, as the roasting level and ripening stage do in CPF obtention [68,69]. Thus, the percentage of addition and the ripening of carobs employed for CPF production may change the final color of the resulting goods [37]. 

In an interesting study by Popov-Raljić et al. (2009) [70], it was found that even after 3 days of storage at room temperature in polyethylene bags, different breads changed color due to staling occurrence; the physical dimensions of the employed flours, and their peculiar composition (i.e., presence of pigments, fibers) were relevant for its development. Our results, despite different formulations, share the trend found by the same authors who had lighter breads at the end of the storage period. In another work, Tsatsaragkou et al. (2017) [54] found that CPF particle size changes the final quality of gluten-free breads. The finer the CPF increments, the darker the breads, and the protein content directly augments the b* value. The same authors also compared the technologies for CPF production, finding that the jet milling technique led to the finest CPF and to darker and browner breads. Of course, the crust is normally darker than the crumbs as it is the primary layer exposed to cooking and, thus, to Maillard reaction occurrence. This trend may be explained by the minimal migration of moisture from the crumb to the crust [70]. This behavior does not directly influence the general color of the entire bread slice.

Generally, as awaited, the storage diminished all the attributes of the bread with respect to flavor and odor, and it was difficult to assess the sourness increment for this type of product, and especially regarding CPF. It is likely that the use of kefir may have enhanced this perception during the storage. The use of protective packaging helped to limit bread decay and to maintain the textural characteristics. It can be said that the limited water mobility produced a firmer bread at the end of the storage, which was perceived by panelists as a less chewy bread.

Considering consumers’ responses, as reported in the results section, the tested product obtained moderate appreciation. These findings are consistent with recent research showing that consumers often give moderate evaluations to new or unfamiliar products that lack distinctive attributes or strong hedonic appeal [71,72].

We noted the ineffectiveness of the information on consumers’ perception of the product. On the contrary, existing literature suggests that providing consumers with information about a product’s health benefits and composition typically enhances its perceived value and sensory evaluation [15,16]

One possible explanation, supported by the literature, could be that the specific health benefits or compositional advantages highlighted were either not compelling enough or not effectively communicated to positively influence consumer perceptions [73].

Siegrist et al. (2008) [74] suggest that consumers’ trust in the source of information and their existing beliefs about food can significantly mediate the impact of health and nutritional information on product evaluations.

Consumers’ pre-existing knowledge and familiarity with specific food attributes or ingredients can significantly impact their acceptance and valuation of new products [22].

This aligns with recent literature advocating for comprehensive nutritional education as a strategy to positively influence consumer choices [75].

The emerging effects of involvement agree with previous research suggesting that higher health involvement leads to greater product appreciation and willingness to pay [16,73]. Consumers with higher involvement in health aspects are more likely to value health-related attributes and are willing to pay a premium for products that meet their health standards [15,16]. The low attention given to the glycemic index specifically could be attributed to a general lack of awareness of this concept among consumers, as highlighted by [16], which found that consumer knowledge significantly impacts food choice behavior.

## 5. Conclusions

This research has elucidated the role of CPF addition in a gluten-free product. Substantial changes in color, aroma, and texture were registered with respect to common gluten-free breads or bread-like products. The packaging protected the snack from exposure to intensive thermal stress, allowing it to maintain its original features. The consumer test conducted in this study provides valuable insights into the impact of information, knowledge, and involvement on the acceptance of and WTP for a carob flour-based bakery product. Both the informed and uninformed groups showed moderate appreciation for the product, with no substantial differences in their evaluations. The provision of detailed information about the product’s composition and health benefits did not significantly enhance the liking or WTP among consumers. Awareness of carob flour is associated with greater WTP for the tested product. Future research should focus on broader demographic studies to capture diverse consumer segments, longitudinal studies to assess the long-term impact of educational interventions on consumer behavior, the examination of other health-oriented aspects of the carob-based products (as the gluten-free aspect), and consumers’ acceptance in different food categories. A possible step to better evaluate information effects could be involving an elderly population segment and/or subjects already suffering from diabetes or other non-communicable diseases connected to diet and nutrition.

## Figures and Tables

**Table 1:** Questions describing the involvement of respondents in the survey.

I consider myself a consumer that pays attention to health aspects
I carefully read the labels
I pay attention to the composition of the product in terms of ingredients
I mind the glycemic index

**Table 2:** Technological properties of carob flour-based bakery product immediately after preparation and packaging.

	Mean ± St. Dev.	Mean ± St. Dev.
Moisture (%)	33.1 ± 0.26	
Water activity	0.927 ± 0.001	
Hardness (N)	161.33 ± 30.57	
Friability (mm^−1^)	0.26 ± 0.07	
	Crumb	Crust
*L*	30.40 ± 1.64	23.92 ± 0.8
a*	9.24 ± 0.53	1.9 ± 0.31
b*	15.71 ± 0.77	1.4 ± 0.47
*C*	18.23 ± 0.9	2.37 ± 0.52
h*	59.55 ± 0.91	35.64 ± 5.48

**Table 3:** Results of sensory qualitative descriptive analysis (QDA) of fresh bakery product (T_0_) and at the end of packaged storage time (T_30_) in ASL condition.

		T0		T30		
		Mean	St. Dev.	Mean	St. Dev.	Sign.
appearance	crumb color intensity	4.89 ± 0.33		4.44 ± 0.73		
	crust color intensity	5.00 ± 0.00		4.22 ± 0.83		*
	air bubbles dispersion	2.56 ± 1.01		1.67 ± 1.32		
odor	cocoa	2.33 ± 1.41		2.89 ± 1.27		
	beans	2.22 ± 1.20		1.33 ± 0.71		
	rye	3.33 ± 1.22		2.33 ± 1.00		
taste	sweet	3.22 ± 1.56		2.56 ± 1.24		
	bitter	2.22 ± 1.20		2.33 ± 1.41		
	sour	2.11 ± 0.93		3.33 ± 1.22		*
	salty	1.78 ± 0.83		1.78 ± 0.97		
flavor	cocoa	3.00 ± 1.66		2.33 ± 1.00		
	gingerbread	2.44 ± 1.24		1.78 ± 1.09		
	sesame	1.78 ± 0.83		1.44 ± 0.73		
	walnut	1.67 ± 0.71		2.00 ± 0.71		
	rye	3.00 ± 1.22		2.56 ± 1.42		
texture	hardness	3.00 ± 1.00		3.00 ± 1.32		
	chewiness	3.11 ± 1.17		1.89 ± 1.16		*
	brittleness	2.22 ± 0.97		2.33 ± 1.12		

Legend: Significant differences between values on the same row are marked by asterisks (*p* < 0.05).

**Table 4:** Results of colorimetric parameters of fresh bakery product (T_0_) and during storage in ASL conditions.

	*L*	a*	b*	*C*	h*
T_0_	30.40 ± 1.64	9.24 ± 0.53 ^b^	15.71 ± 0.77 ^c^	18.23 ± 0.9 ^b^	59.55 ± 0.91
T_7_	29.44 ± 3.24	8.79 ± 0.48 ^b^	14.80 ± 1.45 ^b^	17.03 ± 1.53 ^b^	59.07 ± 1.39
T_14_	29.25 ± 2.19	7.33 ± 0.37 ^a^	12.74 ± 1.04 ^a^	14.70 ± 1.05 ^a^	60.04 ± 1.21
T_30_	28.71 ± 2.71	7.28 ± 0.32 ^a^	12.42 ± 1.05 ^a^	14.33 ± 1.2 ^a^	59.53 ± 1.30
sign.	n.s.	***	***	***	n.s.

Legend: Data followed by different superscript letters in the same row are significantly different (LSD test, *p* < 0.05); asterisks indicate significance at *** *p* < 0.001. n.s., not significant.

**Table 5:** Socio-demographic characteristics of the sample.

		Group A (Not Informed) n = 52	Group B (Full Information) n = 50	Total	Differences between Groups
Gender	Female	61.5%	66.0%	63.7%	n.s.
	Male	38.5%	34.0%	36.3%	
Age (mean values and standard deviation)		25.8 (9.22)	27.8 (9.63)	26.8 (9.4)	n.s.
Education	Undergraduate	55.8%	46.0%	51.0%	n.s.
	Graduate (bachelor’s degree)	26.9%	22.0%	24.5%	
	Graduate (master’s degree or higher education)	17.3%	32.0%	24.5%	
Financial situation	Good	13.5%	24.0%	18.6%	n.s.
	Normal	61.5%	62.0%	61.8%	
	Limited	15.4%	10.0%	12.7%	
	Difficult	3.8%	2.0%	2.9%	
	I’d rather not answer	5.8%	2.0%	3.9%	

n.s. = not significant. Pearson’s chi-square test is used to compare socio-demographic characteristics of respondents regarding gender, education, and financial situation; *t*-test was applied for age.

**Table 6:** Statistical analysis of liking and WTP of the two sub-groups (A = not informed; B = full information).

	Groups	n	Mean ± St. Dev.	Levene Test for Equality of Variances		*t*-Test for Mean Equality	
				F	Sign.	T	Sign.
Overall quality of the product (1—Extremely bad; 5—Neither good nor bad; 9—Very good)	A	52	4.75 ± 2.150	0.355	0.553	−1.090	0.278
	B	50	4.30 ± 2.013				
WTP for 1 kg of the product (Euros)	A	52	1.94 ± 1.614	0.061	0.806	−0.130	0.897
	B	50	1.90 ± 1.669				

**Table 7:** Liking and WTP per knowledge of the meaning of glycemic index.

	Knowledge of Glycemic Index (Yes/No)	N	Mean	St. Dev.	Levene’s Test		*t*-Test	
					F	Sign.	T	Sign. (2-Tailed)
Overall quality of the product (1—Extremely bad; 5—Neither good nor bad; 9—Very good)	Yes	50	4.92	2.009	0.034	0.854	1.878	0.063
	No	52	4.15	2.109				
WTP for 1 kg of the product (Euros)	Yes	50	2.08	1.700	0.300	0.585	0.960	0.339
	No	52	1.77	1.567				

**Table 8:** Liking and WTP per knowledge of carob flour as an ingredient.

	Have You Ever Heard of Carob Flour?	N	Mean	St. Dev.	Levene’s Test		*t*-Test	
					F	Sign.	T	Sign. (2-Tailed)
Overall quality of the product (1—Extremely bad; 5—Neither good nor bad; 9—Very good)	Yes	50	4.88	2.282	1.994	0.161	1.68	0.096
	No	52	4.19	1.837				
WTP for 1 kg of the product (Euros)	Yes	50	2.34 *	1.791	4.542	0.036	2.609	0.010
	No	52	1.52 *	1.365				

* Statistically significant at *p* < 0.05.

**Table 9:** Respondents’ attitudes towards food.

	Mean	St. Dev.
I consider myself a consumer that pays attention to the health aspects	3.80	1.04
I carefully read the labels	3.31	1.20
I pay attention to the composition of the product in terms of ingredients	3.58	1.11
I mind the glycemic index	2.62	1.24
I give priority to the product price	3.34	1.03

**Table 10:** Liking and WTP per high/low-involved groups (1—Extremely bad; 5—Neither good nor bad; 9—Very good).

	Involvement (Groups)	n	Mean	St. Dev.	Levene Test for Equality of Variances		*t*-Test for Mean Equality	
					F	Sign.	T	Sign. (2-Tailed)
Overall quality of the product (1—Extremely bad; 5—Neither good nor bad; 9—Very good)	High	54	4.96 *	2.09	0.286	0.594	−2.272	0.025
	Low	48	4.04 *	1.99				
WTP for 1 kg of the product (Euros)	High	54	2.30 *	1.82	6.461	0.013	−2.573 ^a^	0.012
	Low	48	1.50 *	1.29				

^a^ Welch’s correction was applied. * Statistically significant at *p* < 0.05.

## Data Availability

The original contributions presented in the study are included in the article, further inquiries can be directed to the corresponding author.
